# Calcium imaging revealed no modulatory effect on odor-evoked responses of the *Drosophila* antennal lobe by two populations of inhibitory local interneurons

**DOI:** 10.1038/s41598-017-08090-y

**Published:** 2017-08-10

**Authors:** Martin F. Strube-Bloss, Veit Grabe, Bill S. Hansson, Silke Sachse

**Affiliations:** 1 Max Planck Institute for Chemical Ecology, Department of Evolutionary Neuroethology, Hans-Knöll-Straße 8, 07745 Jena, Germany; 2 Department of Behavioral Physiology & Sociobiology, Theodor-Boveri-Institute of Bioscience, Biocenter University of Würzburg, Am Hubland, 97074 Würzburg Germany

## Abstract

Although we have considerable knowledge about how odors are represented in the antennal lobe (AL), the insects’ analogue to the olfactory bulb, we still do not fully understand how the different neurons in the AL network contribute to the olfactory code. In *Drosophila melanogaster* we can selectively manipulate specific neuronal populations to elucidate their function in odor processing. Here we silenced the synaptic transmission of two distinct subpopulations of multiglomerular GABAergic local interneurons (LN1 and LN2) using shibire (*shi* ^*ts*^) and analyzed their impact on odor-induced glomerular activity at the AL input and output level. We verified that the employed *shi* ^*ts*^ construct effectively blocked synaptic transmission to the AL when expressed in olfactory sensory neurons. Notably, selective silencing of both LN populations did not significantly affect the odor-evoked activity patterns in the AL. Neither the glomerular input nor the glomerular output activity was modulated in comparison to the parental controls. We therefore conclude that these LN subpopulations, which cover one third of the total LN number, are not predominantly involved in odor identity coding per se. As suggested by their broad innervation patterns and contribution to long-term adaptation, they might contribute to AL–computation on a global and longer time scale.

## Introduction

The survival of most animal species depends on processing of olfactory information from the environment. All animals are surrounded by an incredibly complex odor world comprised of pheromones, food or oviposition cues, as well as attractive or repellent odors that strongly affect behavioral decisions. How the olfactory system encodes the different chemical components to build an internal neural representation of the outer odor world still, remains an open question. A so far well-studied - but yet not fully understood - network model is the first olfactory neuropil of the insect brain, the antennal lobe (AL). The AL represents an analogous organization compared to the vertebrate olfactory bulb (for review cp.^1, 2^). The morphological and functional subunits of the AL, the olfactory glomeruli, receive input via olfactory sensory neurons (OSN) located on the third antennal segment and on the maxillary palp^3^. OSN activity is relayed to projection neurons (PNs) either through direct synaptic connections or via local interneurons (LNs). The PNs transmit AL activity via different neuronal tracts to higher-order processing centers^4^. Different LN types shape the spatio-temporal response pattern of the glomeruli via a complex interplay of temporal excitation and inhibition^5–8^. In honeybees this input-output computation results in a sharpening of responses in order to increase the contrast between different odor stimuli^5^, whereas in *Drosophila melanogaster* a broader odor tuning^9^ and odor dependent modulation could be observed^10^. However, both types of modulation result in an input-output transformation of the olfactory signal in the AL^9^.

The AL of *Drosophila melanogaster* comprises about 200 LNs connecting 54 glomeruli^11–13^. The majority of LNs releases the inhibitory neurotransmitter gamma-amino-butyric-acid (GABA)^6, 14, 15^ or glutamate^16^, but also excitatory, cholinergic LNs are part of the complex AL-network^17^. Several studies have been dedicated to characterize the complex variety of different LN types in *Drosophila* using LN-specific GAL4 lines. These studies could show that LNs can be classified according to glomerulus-specific innervation patterns^12, 15, 18, 19^ as well as intrinsic electrophysiological properties^20^, odor response properties^10^, diversity of neurotransmitter profiles^12^ and a varying sensitivity of the target neurons^8^. A previous study demonstrated that inhibitory LNs of the fly AL are required for behavioral fine discrimination of highly similar odors^21^, suggesting that LNs are involved in modulating the olfactory code in the AL in general.

In order to elucidate the contribution of single LN-types to odor coding and processing, we manipulated two subpopulations of LNs and analyzed the functional consequences in input (OSNs) and output neurons (PNs) of the *Drosophila* AL by imaging calcium dynamics. We used a temperature-sensitive form of shibire (*shi* ^*ts*^) in order to reversibly block synaptic transmission between defined LN-types and their post-synaptic partners. We focused on two LN-populations, which can be selectively labeled by two enhancer trap lines comprising ~60 LNs (LN1-type = *NP1*2*27-GAL4* as well as LN2-type = *NP2426-GAL4*) of about 200 LNs in total and which are characterized as two non-overlapping populations of GABAergic, multiglomerular LNs^15, 20, 22^. Using two different binary transcription systems, we were able to monitor odor-evoked responses in OSNs or PNs by driving expression of the calcium sensor GCaMP under control of the *Orco-* or *GH146-GAL4* line, respectively, while we simultaneously silenced the synaptic transmission of either the LN1- or the LN2-type LN population via expression of *shi* ^*ts*^. To ensure that the employed *shi* ^*ts*^ was functional, we expressed *shi* ^*ts*^ in OSNs using the *Orco-Gal4* promotor line and monitored odor-induced calcium responses at the PN level. Activation of *shi* ^*ts*^ dramatically reduced olfactory input to the AL compared to the parental controls and was therefore efficient to silence synaptic transmission. However, when we silenced synaptic transmission of LN1- or LN2-type LNs using the same *shi* ^*ts*^ construct, neither the odor-induced OSN (input) nor the PN (output) glomerular pattern was affected. We therefore conclude that the two populations of multi-glomerular GABAergic LNs targeted in our study are not predominantly involved in odor coding per se. Our results in combination with the widely spread morphological innervation patterns and the fact that both LN types contribute to long-term central adaptation effects^22–24^ rather suggest a more general function of LN1- and LN2-type neurons regarding global inhibition and modulating AL–computation on a longer time scale.

## Materials and Methods

### Fly Lines

All fly stocks were maintained on conventional cornmeal-agar-molasses medium under a 12 hr light:12 hr dark cycle at 23 °C to prevent activation of *shi* ^*ts*^ prior to the experiments. Transgenic fly lines (Bloomington Stock Center; http://flystocks.bio.indiana.edu/) used were as follows: *GH146-LexA* ^19^, *LexAop-GCaMP1.6*, *LexAop-GCaMP3.0*, *Orco-LexA* ^25^, *NP1227-GAL4*, *NP2426-GAL4* ^15^ and *UAS-shi* ^*ts*^ ^26^.

### Electroantennograms

Female wild type flies were immobilized on ice and placed in a pipet tip exposing the fly’s head through the tip opening. Two glass capillaries equipped with a silver wire in a silver-chloride solution were used to record antennal activity. One electrode inserted into the eye served as reference electrode. The recording electrode was connected to an EAG-probe (Syntech, Germany) and 10x pre-amplified. The electrode tip was attached onto the 3^rd^ antennal segment. The recorded signal was further amplified by a high impedance DC-Amplifier and digitally converted with a sampling rate of 10 kHz (IDAC-4 USB, Syntech) using a dedicated software (GC-EAD, Syntech, Germany).

### Optical Imaging

The *in vivo* fly dissection was carried out as previously described^27^ in saline (130 mM NaCl, 5 mM KCl, 2 mM MgCl_2_, 2 mM CaCl_2_, 36 mM saccharose and 5 mM Hepes) at pH 7.3 adjusted with 1 M NaOH. Additionally a thermosensory probe (thermometer 307, B + B Thermo Technik GmbH, Donaueschingen, BW, Germany) was attached to the dissection chamber detecting the temperature of the saline. Imaging measurements were acquired using a CCD-camera (pro. imaging, sensi cam) attached to an upright fluorescent microscope (Olympus BX51WI) which was controlled via the software TILL visION (TILL Photonics, Germany). Excitation of the calcium-sensitive protein GCaMP1.6 or GCaMP3.0 (see fly lines) was provided via a Polychrome V (TILL Photonics, Germany). The imaging protocol lasted for 40 frames at 4 Hz with a stimulus duration of 2 s. Temperature increase of the flies was achieved using a circular punched copper plate, coated with a two-component silicone (KwikSil, WPI, www.wpiinc.com) to prevent conductance, connected to a soldering iron and placed between the fly and the objective. The copper plate was heated through a voltage of 2.5 V applied to the soldering iron giving us a constant saline temperature of 31 °C during the experiment.

### Odor stimulation

The stimulation during EAG as well as during the optical imaging procedure was applied using a stimulus controller (Syntech Stimulus Controller CS-55, Germany) generating a continuous air flow of 1.0 lpm added to a stimulus flow of 0.5 lpm which was shifted between a blank and a stimulus pipette to prevent mechanical stimulation. The odors (6 µl, all odors from Sigma Aldrich) were diluted in mineral oil, pipetted on a circular filter paper and placed in Pasteur pipettes. These were then attached to the tubing of the stimulus controller.

### Identification of glomeruli

Odor evoked spatiotemporal response patterns in the imaged focal plane of choice were assigned to the corresponding glomeruli using an atlas of the *in vivo* AL^11^. Glomerular innervations in the AL of the two used GAL4-lines, *Orco-GAL4* and *GH146-GAL4*, are already well characterized^11^. Initial landmark glomeruli were identified based on their known response profile to the used odors (e.g. isoamylacetate – DM2, benzaldehyde – DL5^28^) or their prominent morphology (e.g. DA1, VA1d and VA2). All remaining glomeruli were identified by their close vicinity to the landmark glomeruli according to their stereotypic neighboring orientation (e.g. DA1 – DA4l and VA6; DL5 – DC1 and DL1; DM2 – DM1, DM3, DM5, DM6; VA1d – VA7l; VA2 – VM2). Only glomeruli which could be reliably identified subsequently in every specimen were taken into account for further statistical analysis.

### Data analysis

Calcium signals were analyzed with custom-written programs in IDL 6.4 (ITT Visual Information Solutions). Beginning with a background, bleach and movement correction to minimize artifacts and continuing with identification of the observed glomeruli, precise response kinetics (ΔF/F) for each glomerulus were calculated^27^. Further data analysis was carried out using Matlab. To apply principal component analysis (PCA) and to calculate Euclidean distances, EAG as well as imaging data were organized in stimulus dependent population vectors in the following way: For a given stimulus *a* and a population of *n* recordings (EAGs) or glomeruli (imaging) we constructed the *n*-dimensional signal (voltage or deltaF/F) vector *v* ^*a*^ at each point in time. These vectors were used in different combinations for principal component analysis (PCA) and calculation of time-resolved Euclidean distance (L^2^-Norm) for different pairs of rate vectors (*v* − *v* ^*b*^) in the following way *d*(*t*) = (*Σ*(*v*_*i*_^*a*^(*t*) − *v*_*i*_^*b*^(*t*))^2^)^*1*/*2*^.

### Statistical analyses

To test for statistical significance we compared the mean of the stimulus-dependent maximal fluorescence distributions using a balanced one-way ANOVA. To test for statistical differences of the Euclidean distances and differences in the averaged maximal rates, a two-sided Wilcoxon rank sum test was performed.

## Results

**Figure 1:**
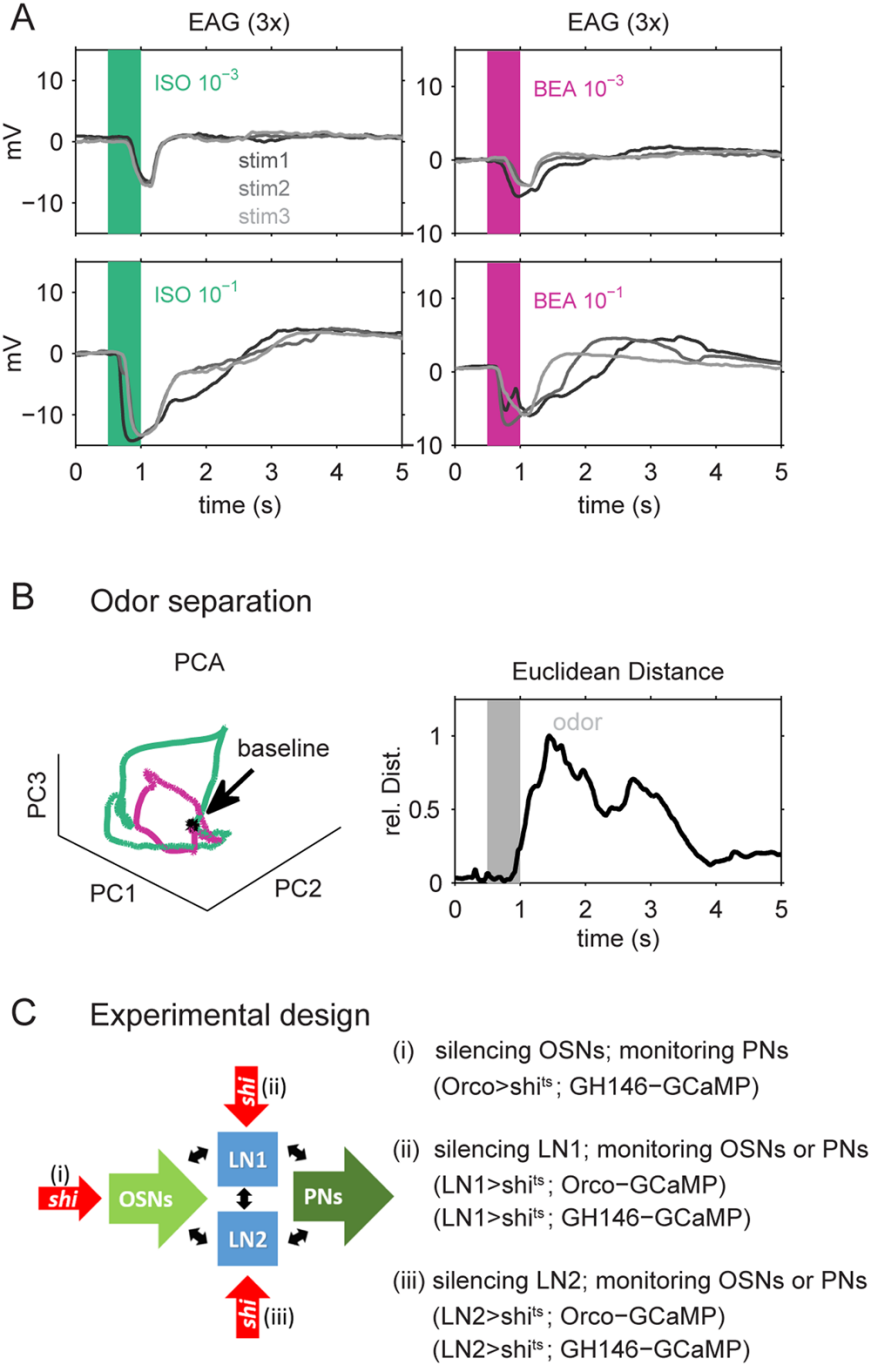
Odors can be separated based on electro-antennogramm (EAG) responses. The two odors isoamylacetate (ISO, green) and benzaldehyde (BEA, violet) can be well separated at the level of the sensillum-population activity. (**A**) EAG responses during application of ISO and BEA. Each odor stimulus is repeated three times (steps of grey) at a low (0.001 vol/vol; upper two panels) and high concentration (0.1 vol/vol; lower two panels). The odor stimulation (500 ms) is indicated by colored bars in each panel. All stimuli evoked a reliable and reproducible EAG response reflecting naturally stimulation conditions in the antennal working range. (**B**) The three stimulus repetitions in 10 animals were used to create a population vector (cp. Methods). Principal component analysis of these vectors (left) illustrate that ISO (green) and BEA (violet) induced distinct trajectories during odor application. The black dot refers to the baseline activity before odor onset. The separation of both odors could be verified by their Euclidean distances (right). (**C**) Experimental design: We used two binary transcription systems (LexA-LexAop and GAL4-UAS) to selectively express a temperature-sensitive form of *shibire* (*shi* ^*ts*^) to silence one specific type of neuronal population, while we expressed the calcium sensor GCaMP in neurons that we aimed to monitor. First, we expressed *shi* ^*ts*^ in olfactory sensory neurons (OSN) and GCaMP3.0 in projection neurons (PN) and established the necessary experimental conditions (i). In the next step we expressed *shi* ^*ts*^ in local interneurons type 1 (LN1) and monitored either the OSN or the PN activity, respectively (ii). Furthermore we expressed *shi* ^*ts*^ in local interneurons type 2 (LN2) and monitored again the OSN and the PN activity, respectively (iii).

To ensure that the odors we used for our study can be detected by the peripheral olfactory organs and induce clear odor-evoked activity in the working range of OSNs, we recorded the overall receptor activity using electroantennography (EAG) in wild type flies. We presented the two odors isoamyl acetate and benzaldehyde. Both odors evoked a reliable EAG activity at low (10^−3^) and high (10^−1^) concentrations (Fig. 1A). The overall OSN population activity allowed a reliable stimulus separation already at the antennal level (Fig. 1B), since both odors activate different sets of OSNs and therefore lead to a distinct glomerular activation at the AL level^29^.

To investigate the influence of LN processing onto the activity patterns at the glomerular input and output level, we expressed the calcium sensor GCaMP in either olfactory sensory neurons (OSN, input) or projection neurons (PN, output). A detailed scheme of all genetic manipulations for the different experiments is given in Fig. 1C.

### Silencing synaptic output of OSNs

**Figure 2:**
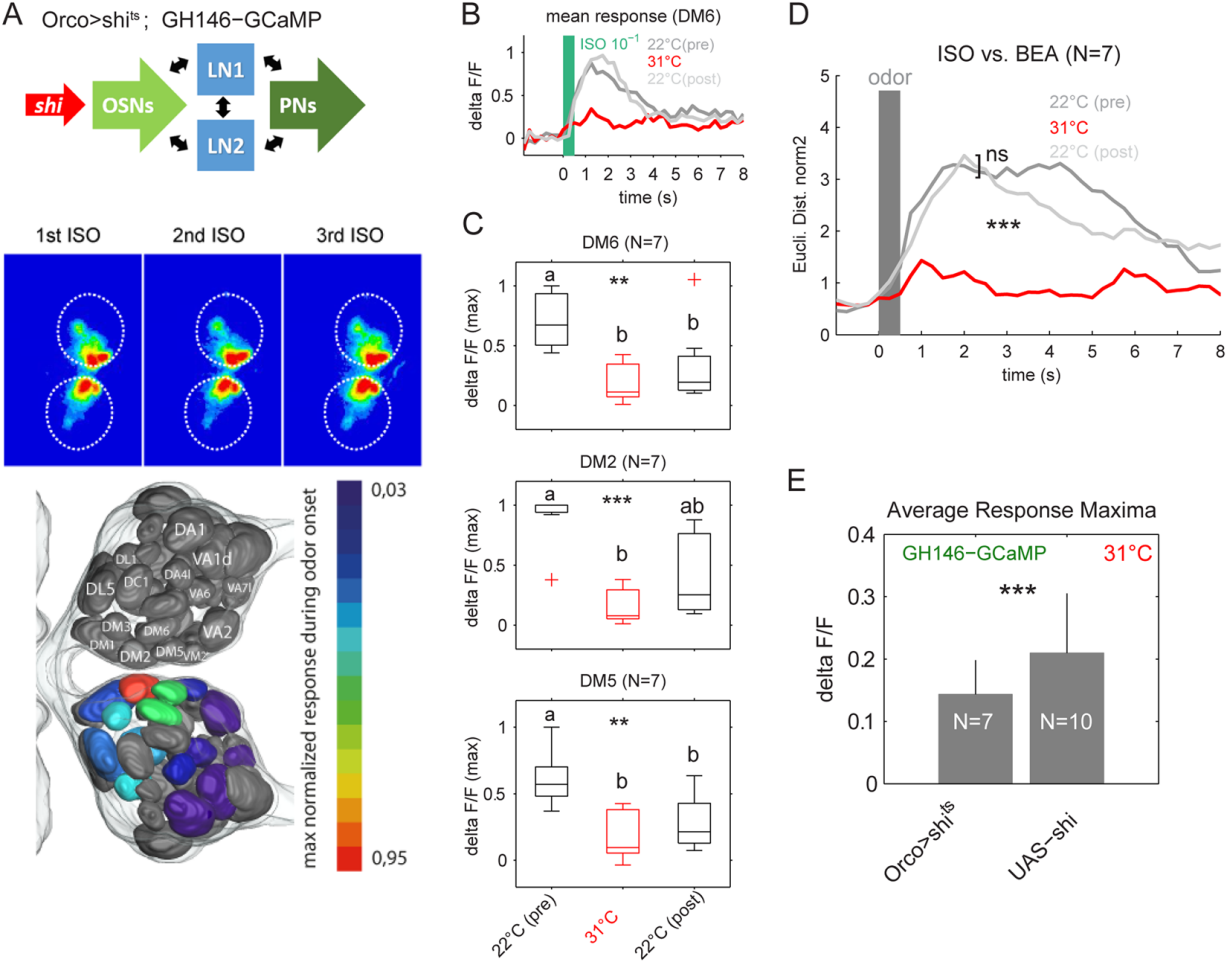
Silencing OSN output. (**A**) We silenced synaptic transmission of OSNs by expressing *shi* ^*ts*^ under the control of the Orco-promotor and monitored calcium signals via GCaMP3.0 in PNs using the GH146 enhancer trap line (see scheme part (i) in Fig. 1). False color coded pictures illustrate odor-evoked calcium responses of PNs during three repetitions of isoamylacetate (1^st^–3^rd^ ISO) as it was performed in each experimental phase. We used the *in vivo* atlas^11^ to identify the same set of 15 glomeruli in every animal (lower panel). (**B**) The responses to the three odor repetitions of each experimental phase were averaged for each glomerulus (DM6 is shown as an example). Phase 1: imaging at the permissive temperature (22 °C (pre), *shi* ^*ts*^ inactive, dark grey), phase 2: imaging at the non-permissive temperature (31 °C, silencing OSNs, red) and phase 3: imaging at the permissive temperature after 10 minutes (22 °C (post), *shi* ^*ts*^ inactive, light grey). Odor stimulation starts at time 0 and lasts for 500 ms (green bar). (**C**) Fluorescence maxima during application of ISO are shown for the three most responsive glomeruli for all three experimental phases (22 °C (pre) = black, 31 °C = red, 22 °C (post) = black) (N = 7). Comparing the experimental phases revealed a significant change in all three glomeruli (ANOVA, **p < 0.01, ***p < 0.001). A pairwise post-hoc Wilcoxon rank sum test (p < 0.05) revealed significant decrease during 31 °C for all three glomeruli. Different letters indicate significant differences. (**D**) Euclidean distances were calculated between the time resolved population vectors of ISO and BEA in each experimental phase. Both odors could be equally well separated before and after silencing the OSNs (Wilcoxon rank sum test, n.s.), whereas odor separation is significantly decreased (collapsed) during the non-permissive temperature (Wilcoxon rank sum test, ***p < 0.001). (**E**) Averaged fluorescence maxima for both odors ISO and BEA pooled during the non-permissive temperature (31 °C) revealed a significant activity decrease compared to the parental control flies (Wilcoxon rank sum test, ***p < 0.001).

Before targeting the two different populations of LNs, namely LN1- and LN2-type neurons, we first established a protocol to reliably and reversibly silence synaptic transmission via a temperature-sensitive form of *shibire* (*shi* ^*ts*^). To do so, we expressed *shi* ^*ts*^ in OSNs to silence their synaptic output and measured odor-evoked calcium signals in PNs (Fig. 1C(i)). We established a reliable approach to measure the same animal in three subsequent experimental phases: phase 1 represents the initial test phase at the permissive temperature (i.e. 22 °C, pre), phase 2 represents the silencing phase at the non-permissive temperature (i.e. 31 °C), and phase 3 represents the recovery phase at the permissive temperature (i.e. 22 °C, post). Here and in all following experiments, we identified the same set of 15 glomeruli in every animal using the *in vivo* 3D *Drosophila* antennal lobe atlas^11^. During the initial test phase at room temperature, we reliably recorded odor-induced signals in all three trials (Fig. 2A). When we increased the temperature in order to block synaptic transmission of OSNs, we observed a clear and strong decrease in glomerular PN activity (Fig. 2B), which was significant for all glomeruli that were mainly activated by the odors tested (Fig. 2C). We observed that the response amplitude in the recovery phase was not entirely reversible and did not reach the same amplitude as during the pre-phase. This is due to the fact that during the duration of an experiment the GCaMP fluorescence is affected by bleaching leading to a general decrease of the GCaMP fluorescence and therefore to a reduced signal-to-noise ratio. In addition, it might be possible that the temperature increase had a prolonged effect on the neuronal conditions causing decreased neuronal responses.

To analyze if the observed activity decrease had an influence on the odor separation between isoamyl acetate and benzaldehyde, we calculated the Euclidean distances (ED) between the odor-induced population activities of the 15 identified glomeruli (Fig. 2D). Both odors could be well separated in the initial test phase and after recovering from the non-permissive temperature, whereas odor separation was significantly reduced when the OSN output was silenced (Wilcoxon rank sum test, ***p < 0.001).

### Temperature increase to 31 °C decreased calcium signals in general

**Figure 3:**
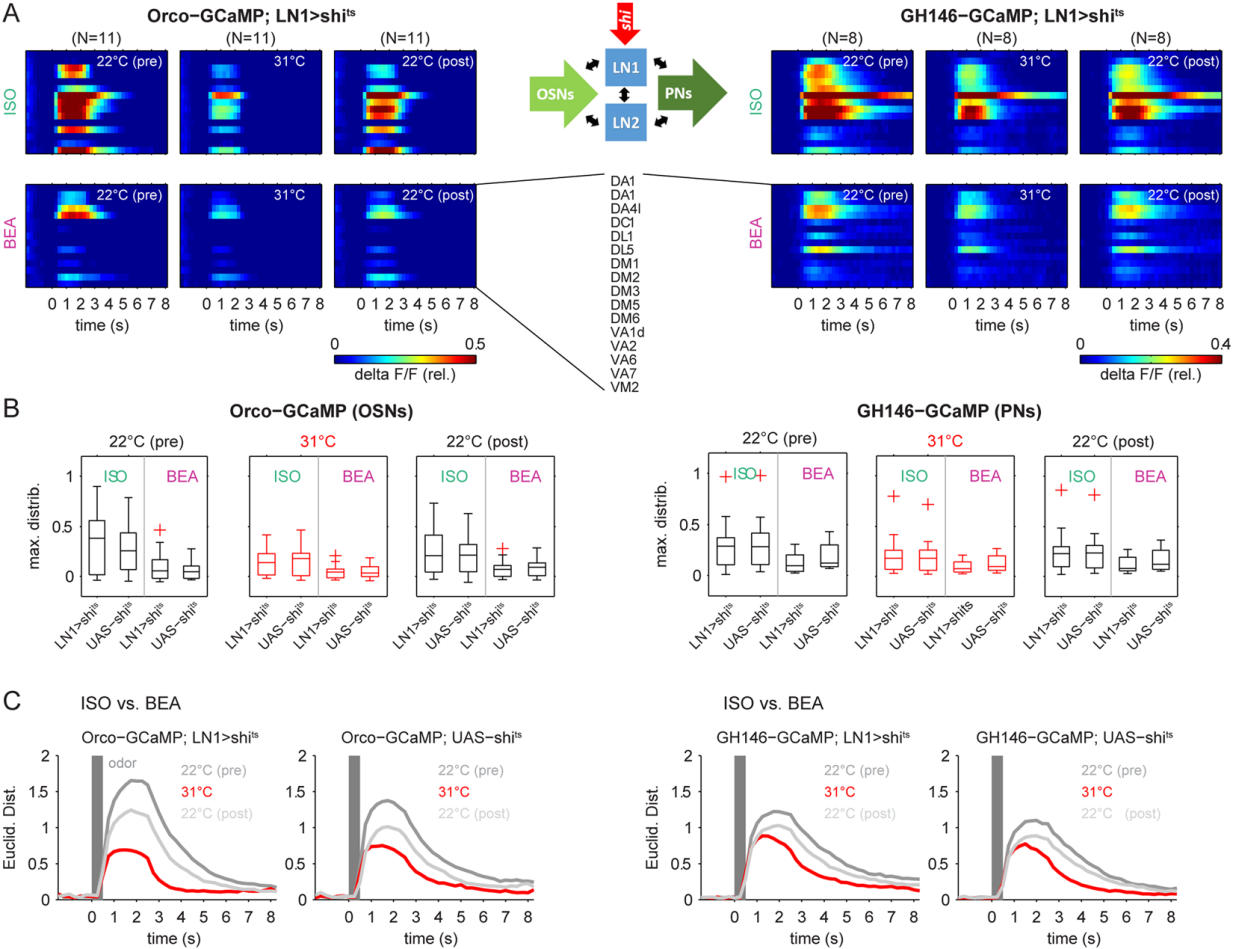
Silencing synaptic transmission of local interneurons type 1 (LN1). (**A**) Synaptic transmission of LN1 was silenced by expressing *shi* ^*ts*^ under the control of the enhancer trap line *NP1227-GAL4* while calcium signals were monitored in OSNs (left: Orco) and PNs (right: GH146, see scheme part (ii) in Fig. 1 for details). False color coded mean activity of 15 glomeruli for the three experimental phases during the presentation of ISO (upper panels) and BEA (lower panels). (**B**) Comparison of the odor-evoked maximal fluorescence distributions between the parental control (UAS-*shi*) and the experimental line (LN1 > shi^ts^) reveals no significant differences between the permissive and non-permissive temperatures, neither for the OSN nor for the PN activity (ANOVA, p > 0.05). (**C**) The odor evoked population vector activity (shown in A) was used to calculate Euclidean distances between both odor representations separately in every experimental phase. The temperature increase to 31 °C led to a drastic decrease in the Euclidean distances (red) in OSN (left) as well as in PN activity (right). At the permissive temperature during the recovery phase, the Euclidean distances increased again but did not completely recover. However, we observed the same effect for the parental control (UAS-*shi* OSNs, N = 9; UAS-*shi* PNs, N = 10).

**Figure 4:**
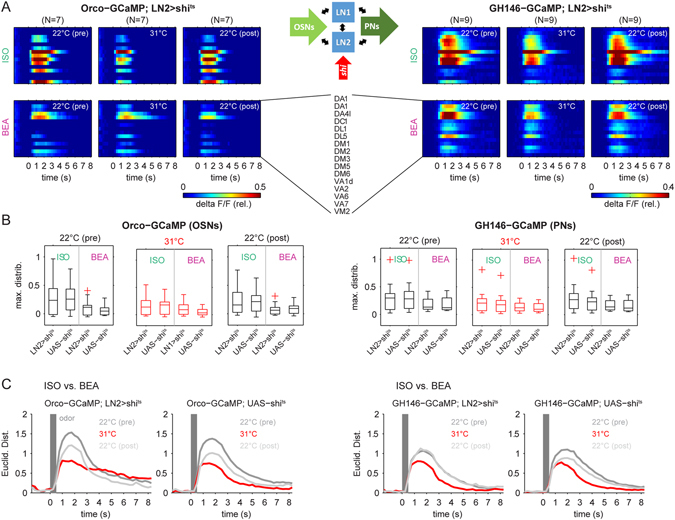
Silencing synaptic transmission of local interneurons type 2 (LN2). (**A**) Synaptic transmission of LN2 was silenced by expressing *shi* ^*ts*^ under the control of the enhancer trap line *NP2426-GAL4* while calcium signals were monitored in OSNs (left: Orco) and PNs (right: GH146, see scheme part (iii) in Fig. 1 for details). False color coded mean activity of 15 glomeruli for the three experimental phases during the presentation of ISO (upper panels) and BEA (lower panels). (**B**) Comparison of the odor-evoked maximal fluorescence distributions between the parental control (UAS-*shi*) and the experimental line (LN2 > shi^ts^) reveals no significant differences between the permissive and non-permissive temperatures, neither for the OSN nor for the PN activity (ANOVA, p > 0.05). (**C**) The odor evoked population vector activity (shown in A) was used to calculate Euclidean distances between both odor representations separately in every experimental phase. The temperature increase to 31 °C led to a drastic decrease in the Euclidean distances (red) in OSN (left) as well as in PN activity (right). At the permissive temperature during the recovery phase, the Euclidean distances increased again but did not completely recover. However, we observed the same effect for the parental control (UAS-*shi* OSNs, N = 9; UAS-*shi* PNs, N = 10).

To initiate expression of *shi* ^*ts*^ the temperature of the fly brain had to be increased from room temperature (22 °C) to the non-permissive temperature of 31 °C. When we established the experimental procedure, we observed that this temperature shift was accompanied by a general decrease in the calcium signal also in control flies (Figs 3C and 4C). Thus the temperature increase itself affected the fluorescence of GCaMP by at least 50%, an effect which was so far only described for mammalian pyramidal neurons^30^. However, although the decrease in GCaMP fluorescence was reasonably strong, we were still able to record and visualize odor-induced glomerular activities in control flies at the non-permissive temperature (Fig. 2E). Most importantly, the reduction of odor-evoked calcium signals was significantly stronger in flies where the synaptic output of OSNs had been silenced with *shi* ^*ts*^ compared to parental controls (Fig. 2E). In conclusion this means that the odor-evoked responses of PNs were nearly abolished in experimental flies, while we were still able to monitor responses in control flies during increased temperature. Hence, these control experiments demonstrate that our experimental approach allows us to effectively silence synaptic output and to monitor the functional consequences by calcium imaging in order to analyze the impact of two different LN subpopulations onto the odor-evoked activity patterns within the AL.

### Silencing synaptic output of LN1

Having established the necessary tools we proceeded to silence the synaptic output of LN1-type neurons by *shi* ^*ts*^ expression (Fig. 1C(ii)). We then monitored odor-evoked calcium signals at both the OSN- and the PN-level, respectively. When testing the parental control flies we observed, as expected, the same reduction of the calcium signal as we detected in our initial experiments (Fig. 2). However, as demonstrated above, the decrease in GCaMP fluorescence still enabled us to monitor and analyze the odor induced glomerular activity patterns in the AL at the non-permissive temperature. In the experimental lines, which expressed *shi* ^*ts*^ in LN1-type neurons, we expected to observe an increase in the odor-induced calcium signals during the non-permissive temperature, since LNs of type 1 have been shown to be GABAergic and to facilitate inhibition^15, 20, 31^. However, contrary to our expectations, we observed a reduction of the odor-induced calcium signals in both OSNs and PNs (Fig. 3A), which was not statistically different from the fluorescence reduction in control flies (Fig. 3B; Supplemental Figure S2). Hence silencing the output of LN1-neurons did not affect the odor-evoked patterns neither at the input, nor at the output level of the AL. To test whether the signal reduction had an influence on the odor separation, we calculated the Euclidean distances between the glomerular activity induced by isoamyl acetate and benzaldehyde. Interestingly, although the Euclidean distances were decreased, the two odors could still be separated when LNs of type 1 were silenced. Both, the experimental line (i.e. *shi* ^*ts*^ expression in LN1-type neurons) as well as the parental control flies showed a similar decrease in the Euclidean distances during the non-permissive temperature (Fig. 3C).

### Silencing synaptic output of LN2

In the next set of experiments, *shi* ^*ts*^ was expressed in LN2-type neurons (Fig. 1C(iii)). The calcium signals were measured again either at the OSN- (input) or at the PN- (output) level. At both processing levels activation of *shi* ^*ts*^ in LN2-type neurons revealed again a reduction of the odor-induced calcium signal to the same amount as in the respective parental control flies (Fig. 4A and B). Furthermore, there was no statistical difference in the averaged odor-induced calcium responses between the LN2 silenced and the parental control flies during the non-permissive temperature (Supplemental Figure S2). Neither the input nor the output glomerular activity of the AL was significantly affected. We again analyzed the odor separation between isoamyl acetate and benzaldehyde in the population of the 15 identified glomeruli by calculating the Euclidean distances in each experimental phase. The initiation of *shi* ^*ts*^ expression during the non-permissive temperature decreased the odor separation at the OSN as well as at the PN level. However, the same effect could be observed in parental control flies (Fig. 4C). We therefore conclude that also silencing the synaptic output of LNs of type 2 had no effect on the glomerular calcium activity, neither at the input nor at the output level of the AL.

### Silencing synaptic output of LN1 and LN2 had also no effect on the spatial glomerular OSN and PN pattern

So far we focused our analysis on the temporal evolution of the calcium signal. However, silencing of LN1-type or LN2-type neurons might have affected the spatial glomerular activity patterns at either the input or the output level of the AL. A possible scenario could be that an initially inhibited glomerulus becomes activated since we release this glomerulus from an inhibitory synaptic output of LN1- or LN2-type neurons. To test for this, we applied a principal component analysis (PCA) on each of the four datasets and ordered the identified glomeruli according to their main contributions (factor loading) to the variation of principal component 1 (PC1). A glomerulus which had most influence on the variation in the odor response over the three experimental phases should be indicated by the highest factor loading. In the extreme such a glomerulus could be active in one experimental phase and inactive in another. However, the sequence of glomeruli we obtained after ranking was not influenced by any treatment. Independent which LN type we silenced, the same glomerular activity pattern was evoked in all three experimental phases. Hence, there was not a single glomerulus added during the non-permissive temperature. Moreover, isoamyl acetate and benzaldehyde induced a distinct spatial activity pattern at both processing levels (OSNs and PNs) as illustrated in the sequence of activated glomeruli. Furthermore, the same odor component evoked a different glomerular sequence depending on the processing level. This difference between the odor-induced OSN and PN glomerular activity pattern is consistent with the literature and represents the input-output transfer of olfactory information by the AL-network^32^.

**Figure 5:**
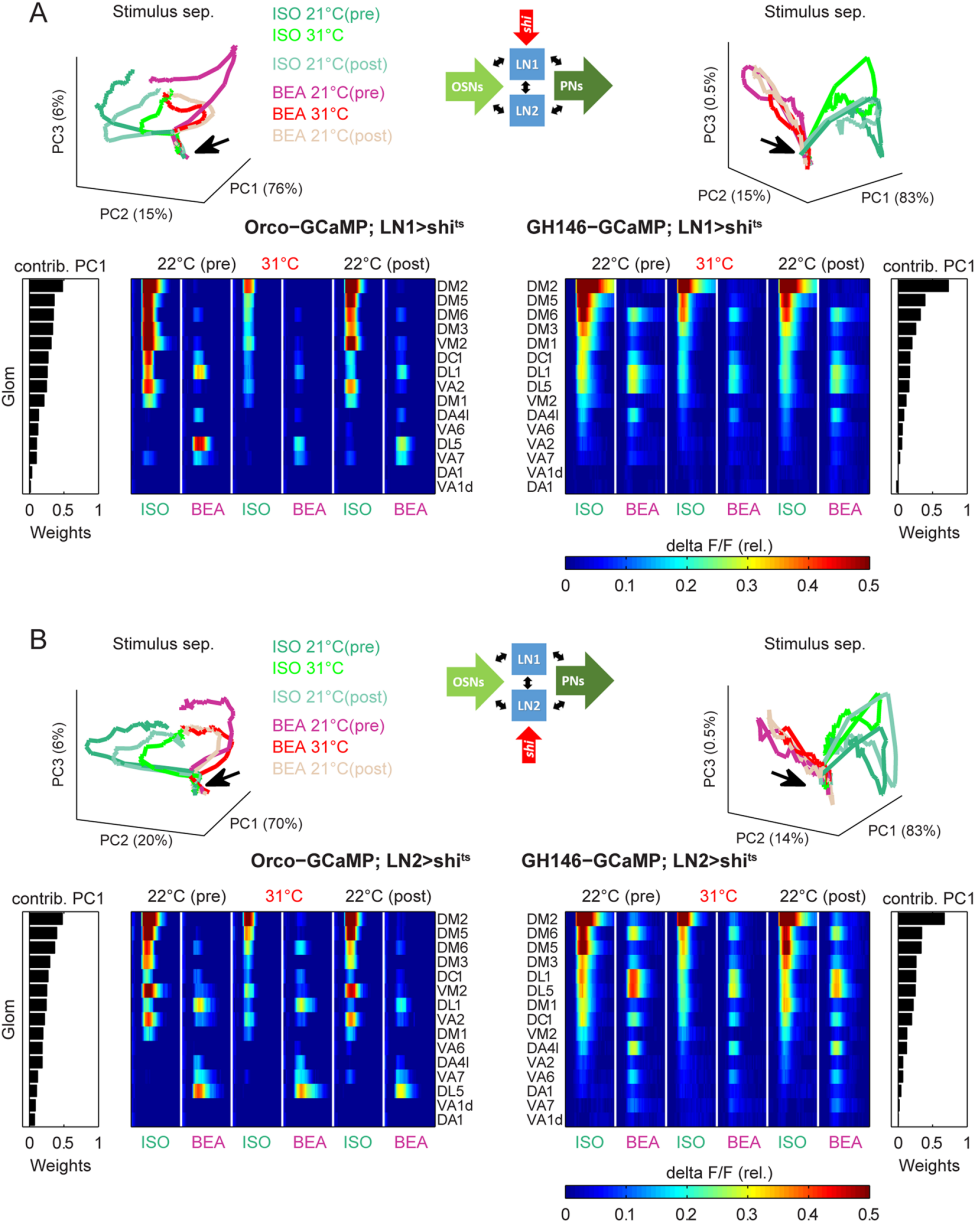
Silencing synaptic transmission of LN1 or LN2 has no influence on the spatial glomerular activity. (**A**) Principal component analysis of odor-evoked calcium responses of flies with silenced LN1-output for the OSN (left) and the PN level (right). Trajectory plots of the first three principal components (PC1, 2 and 3) show a clear separation of ISO and BEA in all experimental phases at both processing levels. The variation explained by the individual PCs is given at the axes. Black arrows mark the baseline activity before odor onset. During temperature increase (silencing LN1-output) the trajectories are strongly collapsed at the OSN level. The factor loadings (weights) of the single glomeruli were used to order them with respect to their contribution to the variability of PC1 for the OSN activity (most left) and the PN activity (most right). The false color coded matrix including the averaged mean fluorescence change is shown for both processing levels with respect to this ordering. The spatial activity patterns at both processing levels (OSNs and PN) were not affected by the temperature shift. However, the calcium signal strength decreased strongly during the temperature increase and recovered during the permissive temperature in the recovery phase (22 °C (post)). (**B**) Same representation as in A for flies with silenced LN2-output.

Taken together, our results demonstrate that the spatial glomerular activity patterns at the AL-input level as well as at the AL-output level are not influenced by silencing LN1- or LN2-type interneurons to the tested odors. Furthermore, the odor-induced trajectories in the principal component space, spanned by the first three principal components (PC1, PC2 and PC3), illustrates a separation of both odors in each experiment and experimental phase. However, this separation is merely due to a general decrease in GCaMP fluorescence, which can be observed at the non-permissive temperature (Fig. 5A and B).

## Discussion

To elucidate the contribution of different LN-types on odor coding and processing, we manipulated two subpopulations of LNs via *shi* ^*ts*^ and analyzed the functional consequences in OSNs and PNs of the *Drosophila* AL using functional calcium imaging. We verified that the employed *shi* ^*ts*^ construct was efficient in blocking synaptic transmission to the AL when expressed in OSNs. Notably, selective silencing of both LN populations did not significantly affect the odor-evoked activity patterns in the AL of the odors tested. Neither the glomerular input nor the glomerular output activity was modulated in comparison to the parental controls. In addition, we observed that the signal amplitude of the odor-evoked responses was strongly affected by the temperature increase.

### LN1- and LN2-type neurons are not predominantly involved in innate odor coding

We focused our study on two types of local interneurons which cover approximately one third of all LNs in the fly AL and which have been morphologically and functionally well characterized in previous studies^15, 20, 31^. Both LN classes innervate homogenously almost all glomeruli^20^, where their presynaptic sites are homogenously distributed throughout the entire AL^15^. In addition, 95% of both LN types have been shown to be GABA-immunoreactive^15, 20, 31^ demonstrating that they release GABA as their inhibitory neurotransmitter. If these LN neurons contribute to the immediate AL odor computation, silencing synaptic transmission of one or the other LN type should directly increase the glomerular calcium activity upon odor stimulation in one or the other glomerulus. However, we observed the opposite effect, i.e. a decrease in activity, independent on the LN type we silenced and independent on the processing level (i.e. OSN- or PN-level) that we monitored during functional imaging. Since this effect can be related to the general fluorescence decrease of GCaMP during the temperature increase^30^ and since it was not significantly different from the fluorescence decrease we observed in the control flies, we conclude that silencing synaptic transmission of LN1- and LN2-type LNs has no effect on the innate odor-induced OSN as well as the PN glomerular calcium activity and therefore odor coding per se for the odors tested. However, a previous study provided evidence that these two LN populations are required for behavioral fine discrimination of highly similar odors following differential conditioning^21^. Since our odors activate completely different sets of OSNs and therefore PNs^29^, a local computation to enhance odor discrimination is therefore not necessary and might be less evident for odors that are perceived differently. However, one has to keep in mind that we tested the innate responses in naïve animals. Hence, learning-induced modulations of neural representations of similar odors by LNs, as demonstrated previously^21^, should not occur. It is conceivable that both LNs types contribute to modulate odor-evoked PN responses to mixtures, where inhibitory interactions have been observed^32^. In addition, LN1- as well as LN2-type neurons have been shown to be involved in the modulation of AL activity on a longer time scale reflecting learning and experience related plasticity^22–24, 33^. Hence both LN types facilitate long-term central adaptation effects and might not contribute to immediate and general coding properties of the AL network, which would be in line with our results.

### Calcium imaging might be too slow to observe the influence of LN1 and LN2 on glomerular AL-activity

Encoding olfactory stimuli in the AL and providing that information to downstream neuronal networks such as the mushroom body calyx or the lateral horn is a rapid process, whereby incoming odor information is transformed into a series of quickly evolving patterns of PN activity representing the AL output^7, 20, 34^. This process facilitates a very fast maximal odor separation at the mushroom body output, two synapses further. In honeybees this fast stimulus separation at the mushroom body output is already established at ~80–120 ms after odor onset^35^. After the animals had built an odor reward association the response to encode the reward associated stimulus at the mushroom body output was shifted by another ~80–100 ms reaching its maximum ~200 ms after odor onset^36^ but still enormously fast. It is therefore conceivable that the AL network including LN processing is tuned to optimally and rapidly extract behaviorally relevant stimuli approximately between 0 and 200 ms after odor onset. In the present study we visualized the intracellular calcium signal with a temporal resolution of 4 Hz which might have been too slow to observe an effect of LN1- or LN2-type neurons on the rapid evolution of odor representation in the AL. Moreover, LN2-type neurons (labeled by *NP2426-GAL4*) were found to be the source of odor-induced oscillatory activity of ~10 Hz^31^. At the same low firing frequency range^20^ observed strong adaptation properties in LN2-type neurons, which they proposed to be related to their intrinsic property and favorable for generating oscillatory responses. However, we might have blocked this oscillation by silencing LN2-type neurons but our temporal resolution might have been to slow to observe any changes in the intracellular calcium concentration following this time range. We therefore cannot exclude that LN1- and LN2-type neurons are involved in modulating odor-evoked responses in the AL at a fast time scale and contribute to synchronize PN firing patterns as shown in locusts^37^.

### Temperature increase led to a general fluorescent decrease in GCaMP based GECIs

In all flies, including the control lines, we observed a strong decrease in calcium activity during the temperature increase. However, heat application in hematocryal insects increases neural activity and should therefore raise the intracellular calcium concentration. Mao and colleagues^30^ compared fluorescent changes of GCaMP based genetically engineered calcium indicators (GECI) in hippocampal pyramidal cells in cell culture at room temperature (i.e. 21–24 °C) and under natural conditions (body temperature: 34.5–35.5 °C) and found a temperature-dependent decrease of about 50%. That temperature represents an important parameter in detection of *in vivo* GFP fluorescence has already been shown several years ago^38^. The underlying cause of this temperature dependence might be associated with the folding and/or redox state of GFP within the cells. We therefore assume that we observed a general property of the GECI. Hence, GCaMP may not be the best tool to observe neural activity if the experimental design requires a temperature shift. However, in comparison with an adequate control we could use it successfully to analyze the consequence of silencing OSN activity.

### The necessity of using appropriate tools

We aimed at analyzing the contribution of LN1- and LN2-type neurons on the input as well as on the output activity of the AL upon odor stimulation. We therefore expressed a temperature-sensitive form of shibire (*shi* ^*ts*^) under control of two selective LN-driver lines to be able to compare the representations of odors with or without LN processing. From a theoretical point of view this was a straight forward experimental design. However, besides our observation that LN1- and LN2-type neurons had no influence on the AL-network activity in the way we measured, it is still possible that the drastic decrease in the calcium signal itself masked the effects induced by silencing the different LN-types.

Since many scientists frequently use genetically engineered flies allowing activation or inactivation of target neurons by temperature shifts and visualization techniques, such as multi-photon imaging are very temperature intense and may heat up the tissue, we propose to include a temperature calibration curve besides the common pH rating curve when advertising new GECIs to sensitize scientists and avoid misinterpretation of data.

## Electronic supplementary material

Supplemental Figures S1 and S2
