# Supplementary material for: Calcium imaging revealed no modulatory effect on odor-evoked responses of the Drosophila antennal lobe by two populations of inhibitory local interneurons
Source: Sci Rep. 2017 Aug 10;7:7854. doi: 10.1038/s41598-017-08090-y (PMC5552818; doi:10.1038/s41598-017-08090-y)
Supplement: Supplementary file 1 — Supplemental Figures S1 and S2 [file 41598_2017_8090_MOESM1_ESM.pdf]

Calcium imaging revealed no modulatory effect on odor-evoked responses of the *Drosophila* antennal lobe by two populations of inhibitory local interneurons

Martin F. Strube-Bloss[1,2]*, Veit Grabe[1]*, Bill S. Hansson[1] and Silke Sachse[1#]

Supplementary Information

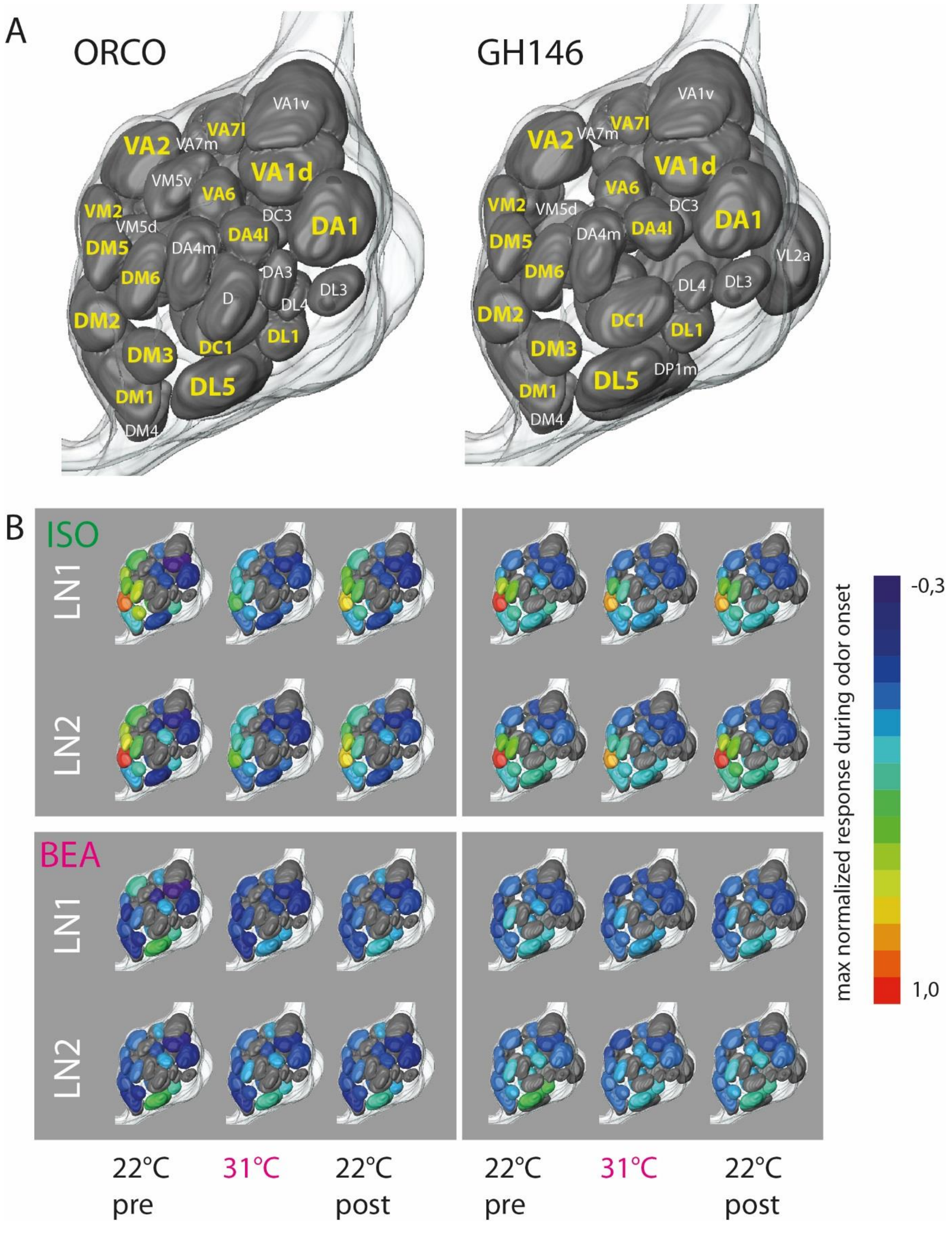

Supplemental Figure S1| Model of the glomerular AL activity pattern. A Spatial maps of all glomeruli labeled by Orco-LexA (left) and GH146-LexA which are mainly overlapping but partially different. The 15 glomeruli with yellow bold labelling are the identified ones for the analysis. Visualization is based on the *in vivo* AL atlas (Grabe et al 2015). **B** Spatial mapping of the maximum responses shown in Fig 5 averaged over all specimens during odor representation of ISO (upper panel) and BEA (lower panel). ORCO (left block) and GH146 (right block) are further split in pre (left), 31°C (center) and post (right) temperature treatment.

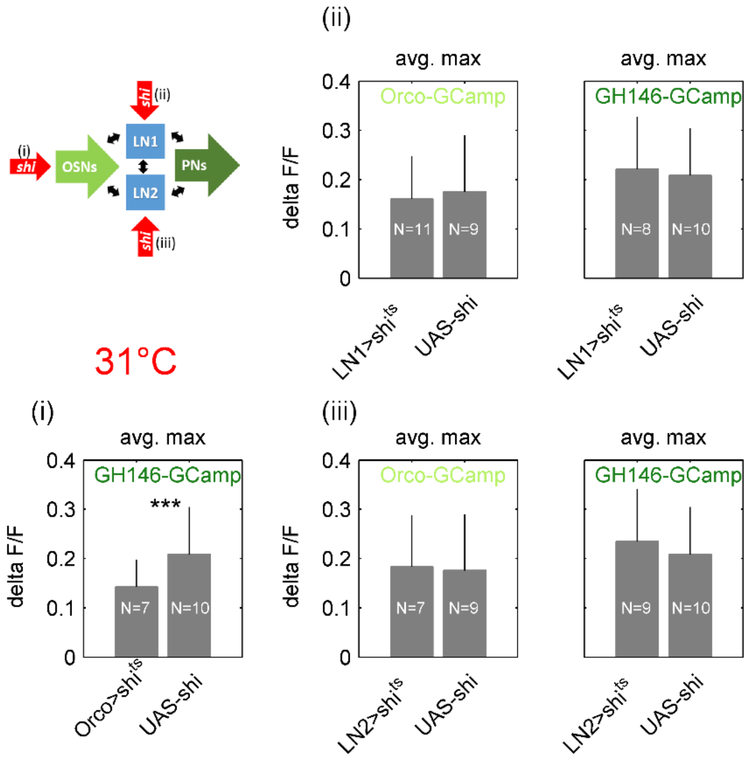

Supplemental Figure S2| Pooled odor-induced activity of the 15 glomeruli during the non-permissive temperature.

We expressed a temperature sensitive form of *shibire* in three different neuronal populations (i=in OSNs; ii=in LN1; iii=in LN2; please see scheme in Figure 1 for details) and compared the pooled odor-induced fluorescence maxima distribution during the non-permissive temperature of 31°C (i.e. silencing synaptic transmission of the treated neurons). Only when we silenced the OSN output (i), we observed a highly significant decrease of the odor-evoked PN activity in comparison to the parental control flies (Wilcoxon rank sum test, ***p<0.001). The odor-evoked OSN as well as the PN responses were not statistically different when the output of LN1 (ii) or LN2 (iii) was silenced.
